# IGFBP5 antisense and short hairpin RNA (shRNA) constructs improve erectile function by inducing cavernosum angiogenesis in diabetic mice

**DOI:** 10.1111/andr.13234

**Published:** 2022-08-07

**Authors:** Jiyeon Ock, Jun‐Kyu Suh, Soon‐Sun Hong, Ju‐Hee Kang, Guo Nan Yin, Ji‐Kan Ryu

**Affiliations:** ^1^ National Research Center for Sexual Medicine and Department of Urology Inha University School of Medicine Incheon Republic of Korea; ^2^ Department of Biomedical Sciences, College of Medicine, and Program in Biomedical Science and Engineering Inha University Incheon Republic of Korea; ^3^ Department of Pharmacology and Medicinal Toxicology Research Center Inha University College of Medicine Incheon Republic of Korea

**Keywords:** angiopathy, diabetes, erectile dysfunction, insulin‐like growth factor binding protein 5, permeability

## Abstract

**Background:**

The incidence of diabetic erectile dysfunction (ED) is rapidly increasing, and due to the severe angiopathy caused by diabetes, current drugs are ineffective at treating ED. Insulin‐like growth factor‐binding protein 5 (IGFBP5) promotes cell death and induces apoptosis in various cell types.

**Objectives:**

To evaluate the effectiveness of IGFBP5 knockdown in improving erectile function in diabetic mice.

**Materials and methods:**

Diabetes was induced by injecting streptozotocin (STZ) intraperitoneally into male 8‐week‐old C57BL/6 mice. Eight weeks after diabetes induction, mice were divided into four groups: a nondiabetic control group and three STZ‐induced diabetic mice groups, which were administered intracavernous injections of phosphate buffered saline, scrambled control shRNA, or shRNA targeting mouse IGFBP5 (shIGFBP5) lentivirus particles. Two weeks later, we measured erectile function by electrically stimulating the bilateral cavernous nerve. To mimic diabetic angiopathy, primary cavernous endothelial cells (MCECs) from healthy mice were cultured and treated with glucose.

**Results:**

IGFBP5 expression in MCECs or cavernous tissues were significantly increased under diabetic conditions, and knockdown of IGFBP5 induced MCECs angiogenic activity under high‐glucose conditions. STZ‐induced diabetic mice had reduced erectile function, but shIGFBP5 treatment resulted in significant improvements (to 90% of the nondiabetic control group level). Furthermore, in diabetic mice, numbers of cavernous endothelial cells, pericytes, and neuronal cells were increased by shIGFBP5 treatment, which also increased eNOS Ser^1177^ phosphorylation, decreased permeability and apoptosis of cavernous endothelial cells. In addition, IGFBP5 was found to mediate the AKT, ERK, p38 signaling pathways.

**Discussion and conclusion:**

Knockdown of IGFBP5 improved erectile function in diabetic mice by promoting cell proliferation and reducing apoptosis and permeability. Local inhibition of IGFBP5 expression may provide a new treatment strategy for diabetic ED and other ischemic vascular or neurological diseases.

## 1 INTRODUCTION

Diabetes mellitus (DM) is a metabolic disease characterized by elevated glucose levels.^1^ Although glycemia is effectively controlled in diabetic patients, complications such as retinopathy, nephropathy, erectile dysfunction (ED), and peripheral neuropathy still occur.^2^, ^3^ ED is one of the main complications of DM and affects more than 50% of all diabetic men.^4^, ^5^ Although the pathogenesis of diabetic ED involves a variety of factors, endothelial dysfunction is considered a key factor.^6^ Oral phosphodiesterase‐5 inhibitors that rely on the bioavailability of endogenous nitric oxide (NO) are currently the first‐line therapy for diabetic ED.^7^ However, patients respond poorly to these medications due to the presence of severe endothelial dysfunction.^8^, ^9^ Therefore, a new strategy is needed that targets damaged endothelial cells.

Endothelial cells play an important role in regulating basal vascular tone and vascular reactivity and are one of the main sources of vasoconstrictor and vasodilator factors.^10^ In corpus cavernosum, endothelial dysfunction is characterized by an imbalance between vasoconstrictors and vasodilators.^11^ In addition, endothelial dysfunction at the interface between blood and peripheral nerves participates in the development of various disease states through different mechanisms, such as by reducing the availability of NO, increasing pro‐inflammatory factor expressions, and changing endothelial permeability.^12^, ^13^ However, the genetic involvement in cavernous endothelial cell dysfunction in diabetic ED mice remains unclear. In a recent study, the physiological and pathological gene expression profiles of mouse cavernous endothelial cells under high glucose (HG) conditions were screened, and many potential treatment candidates for diabetic ED were identified.^14^ Among these candidates, insulin‐like growth factor (IGF) binding protein 5 (IGFBP5) caught our attention because its levels were markedly increased under HG conditions. IGFBP5 is one of six IGFBPs, which by binding with IGF, regulate the mitogenic actions of IGF in a tissue‐ and cell‐dependent manner.^15^, ^16^ IGFBPs can also exert IGF‐independent effects by binding to various receptors.^17^ Of the six IGFBPs, IGFBP5 is most conserved between species and is widely expressed in different cell types.^18^ Furthermore, changes in IGFBP5 levels have been observed in various pathologic conditions. For example, it was shown that increased expression of IGFBP5 in diabetic nerve is associated with the degeneration of motor axons and cell bodies in diabetic neuropathy.^19^ In addition, HG‐induced IGFBP5 expression mediated profibrotic events in cardiac fibroblasts.^20^ However, the effects of IGFBP5 and the mechanisms responsible remain unclear in diabetic ED.

Consequently, the aim of the present study was to determine whether inhibiting the expression of IGFBP5 reduces ED in diabetic mice. We hypothesized that inhibiting the expression of IGFBP5 might promote endothelial cell function, and thus, improve erectile function.

## 2 MATERIALS AND METHODS

### 2.1 Ethics statement and study design

Eight‐week‐old male C57BL/6 mice (weight, 20–25 g; Orient Bio, Inc., Seongnam, Korea) were used in the present study. Animal experiments were performed after obtaining approval from the Institutional Animal Care and Use Subcommittee of our university (approval number: 200309–691). Animal health and behavior were monitored daily, and animals were fed disinfected commercial standard laboratory food and water ad libitum. Mice were maintained at room temperature (RT) (23 ± 2˚C), 40%–60% relative humidity, under a 12‐h light/dark cycle and specific pathogen‐free conditions. Adult male C57BL/6 mice were used for mouse cavernous endothelial cell culture, aortic ring sprouting assays, erectile function evaluation, immunofluorescence staining, and western blot analysis. All animals were anesthetized with ketamine (100 mg/kg) and xylazine (5 mg/kg) intramuscularly (i.m.), and penises were exposed using a sterile technique. Animals were euthanized by 100% CO_2_ gas replacement, which was performed in a closed container at a CO_2_ replacement rate of 10%–30% of container volume/min. Cessation of heartbeat and respiration were confirmed before harvesting tissues. No mouse died during any experimental procedure, and all experiments were performed in a blind manner.

### 2.2 Animals and treatment

DM was induced as previously described.^21^ In total, 70 adult male C57BL/6J mice (8 weeks old) were used in this study (20 for control nondiabetic mice, 50 for STZ‐induced diabetes mice). Briefly, diabetes was induced in 8‐week‐old male C57BL/6 mice by injecting streptozotocin (STZ, 50 mg/kg, i.p., Sigma‐Aldrich, St. Louis, MO, USA) for 5 consecutive days. Eight weeks after STZ injections, only mice with a tail vein blood glucose level higher than 300 mg/dl and significantly decreased body weights were considered to have DM. Fasting and postprandial blood glucose levels were determined with an Accu‐Check blood glucose meter (Roche Diagnostics, Mannheim, Germany). Animals were then anesthetized with ketamine (100 mg/kg) and xylazine (5 mg/kg) i.m., and penises were exposed using sterile technique. Mice were divided into four groups as follows: control nondiabetic mice (*n* = 10) and mice with STZ‐induced diabetes receiving one successive intracavernous injections of phosphate‐buffered saline (*n* = 9, phosphate‐buffered saline [PBS], 20 μl), scrambled control shRNA (*n* = 8, shCon, 5 × 10^4^ infection units in 20 μl) lentivirus particles (Santa Cruz Biotechnology, Dallas, TX, USA) or mouse lentivirus containing shRNA targeting IGFBP5 (*n* = 8, shIGFBP5; 5 × 10^4^ infection units in 20 μl; Santa Cruz Biotechnology) into the midportion of the corpus cavernosum. We performed this experiment twice, once for erectile function and western blot studies and once for histological examination studies. A vascular clamp was used to pressurize the bottom of penises immediately before injection and was left in place for 30 min to restrict blood outflow. This study was repeated twice, once for ICP and western blotting, and once for immunostaining. The sequence of the shRNA targeting mouse IGFBP5 was ACGGCTTATGGGTCATTTA.

### 2.3 Mouse cavernous endothelial cells culture

Primary mouse cavernous endothelial cells (MCECs) were prepared and maintained as previously described.^22^ After sacrifice, penis tissue (*n* = 20) was harvested and transferred to sterile vials containing Hank's balanced salt solution (Gibco, Carlsbad, CA, USA) and then washed twice in PBS (Gibco). Glans penis, urethra, and dorsal neurovascular bundle were then removed. Only corpus cavernosum tissue was used in the study. Corpus cavernosum tissues were cut into ∼1–2 mm sections and covered with Matrigel (Becton Dickinson, Mountain View, CA, USA) in 60‐mm cell culture dishes, and the Matrigel was then polymerized in a 5% CO_2_ atmosphere for 5 min at 37°C. Tissues were cultured with complement medium 199 (M199, Gibco) containing 20% fetal bovine serum (FBS, Gibco), 1% penicillin/streptomycin (Gibco), 0.5 mg/ml heparin (Sigma‐Aldrich), and 5 ng/ml vascular endothelial growth factor (VEGF, R&D Systems Inc., Minneapolis, MN, USA). When cells were confluent (after ∼2 weeks of culture), sprouting cells were used for subcultivation. These cells were seeded into dishes coated with 0.2% gelatin (Sigma‐Aldrich); Cells between passages 2 and 4 were used for all experiments. For the experiments, MCECs were cultured in conditioned medium (CM) under normal glucose (NG, 5 mM glucose, Sigma‐Aldrich) or HG (30 mM glucose, Sigma‐Aldrich) conditions for 72 h at 37°C in a humidified 5% CO_2_ atmosphere.^23^ In order to examine the effect of recombinant mouse IGFBP5 (rmIGFBP5), MCECs were treated with rmIGFBP5 (500 ng/ml; Mybiosource Inc., San Diego, CA, USA) under HG conditions for 72 h.

To characterize cell types, the cells were stained with antibodies to hamster monoclonal anti‐PECAM1 antibody (an endothelial cell marker, 1:50, Millipore, Temecula, CA, USA), rabbit polyclonal anti‐NG2 antibody (a pericyte marker, 1:50, Millipore), mouse monoclonal anti‐CD90 (a fibroblast marker, 1:50, R&D Systems Inc.), rabbit polyclonal anti‐smooth muscle α‐actin (α‐SMA, a smooth muscle cell marker, 1:100, Sigma‐Aldrich), or DAPI (a nucleus marker; Vector Laboratories Inc., Burlingame, CA, USA). Signals were visualized, and digital images were obtained with a confocal fluorescence microscope (K1‐Fluo, Nanoscope Systems, Inc., Daejeon, Korea).

### 2.4 Measurement of erectile function

The penile erectile function was assessed as previously described.^21^ Briefly, bipolar platinum wire electrodes were placed around the cavernous nerve bilaterally, and the nerve was stimulated using 1 or 5 V at 12 Hz and a pulse width of 1 ms for 1 min. Maximal intracavernous pressure (ICP) and total ICP were recorded during stimulation. Total ICP was defined as the area under the curve from cavernous nerve stimulation start until 20 s after stimulus termination. Each electrical stimulus was replicated two times at least at 10‐min intervals. Systemic blood pressure was measured continuously using a noninvasive tail‐cuff system (Visitech Systems, Apex, NC, USA) before ICP measurements. Maximal ICP and total ICP to mean systolic blood pressure (MSBP) ratios were calculated to normalize variations in systemic blood pressure.

### 2.5 Measurement of nitrite oxide levels

The nitrate assay kit (MAK367, Sigma‐Aldrich) was used to determine nitrite oxide (NO) concentration in MCECs and mouse penis tissues (*n* = 16), according to the manufacturer's protocol. For in vitro study, MCECs were seeded in 6‐well plates at a density of 5 × 10^5^ cells/well in 2 ml of M199 medium. After 24 h, MCECs were exposed to glucose conditions with or without IGFBP5 siRNA transfection for 72 h at 37°C in a humidified 5% CO_2_ atmosphere. Then, cultured medium was collected for NO concentration measurement. For in vivo study, the penis cavernous tissues were harvested immediately after ICP functional assessment and stored at −80°C for further NO concentration measurement. NO concentration was measured at a wavelength of 540 nm, using a microplate spectrophotometer (BioTek Instruments Inc., Winooski, VT, USA). Each experiment contained six replicates and repeated four times.

### 2.6 RNA interference

MCECs were serum‐starved for 24 h and transfected with 100 nM of scrambled siRNA control (Sc, Dharmacon, Lafayette, CO, USA) or 100 nM of small interfering RNA (siRNA, Dharmacon) specifically targeting IGFBP5 using Lipofectamine 2000 (Invitrogen, Camarillo, CA, USA) for 5 h in FBS free medium. The medium was then changed to fresh complement medium 199, and cells were maintained in this for 3 days. The sequences of the IGFBP5 siRNA were GAAAGAAAGCAAAGCGUUG, GGAAGGACAGAUAGGAUUA, AGAUGGAGGUCAUUGUAUA, and GGAUAGUACAGUUCAGACA. Experiments were performed 3 days after transfection.

### 2.7 RT‐PCR

Total RNA was extracted from cultured cells using Trizol (Invitrogen) according to the manufacturer's instructions. Reverse transcription was performed using 1 μg of RNA in 20 μl of reaction buffer containing oligo dT primer and AccuPower RT Premix (Bioneer Inc., Daejeon, Korea). The sequences of the primer pairs used were as follows: mouse *IGFBP5*, 5′‐TTG ACC AGC CAG AAC AAA GC‐3′ (forward) and 5′‐CGA CGA GAC CTC TTT CCC TT‐3′ (reverse); *GAPDH*, 5′‐CCA CTG GCG TCT TCA CCA C‐3′ (forward) and 5′‐ CCT GCT TCA CCA CCT TCT TG‐3′ (reverse). PCR was performed using following conditions: 30 s at 94°C (denaturation), 30 s at 60°C (annealing), and 1 min at 72°C (extension) for 30 cycles in a DNA Engine Tetrad Peltier Thermal Cycler. To analyze PCR products, 10 μl of each PCR reaction mix was electrophoresed on 1% agarose gel and detected under ultraviolet light. GAPDH was used as an internal control.

### 2.8 Tube‐formation assay

Tube‐formation assays were performed as previously described.^22^ Briefly, ∼100 μl of growth factor‐reduced Matrigel (Becton Dickinson, Mountain View, CA, USA) was dispensed into 48‐well tissue culture plates at 4°C. After gelling for at least 10 min at 37°C, conditioned MCECs (exposed to glucose conditions with or without rmIGFBP5 or transfection) were seeded onto gels at 1 × 10^5^ cells/well in 300 μl of M199 medium. Tube formation was monitored for 12–16 h, and images were then taken using a phase‐contrast microscope (CKX41, Olympus, Tokyo, Japan). Numbers of master junctions from four separate experiments were quantified using Image J software (National Institutes of Health [NIH] 1.34, http://rsbweb.nih.gov/ij/) in a blinded manner.

### 2.9 Cell migration assay

MCEC migration assays were performed using the SPLScar Block system (SPL life sciences, Pocheon‐si, Gyeonggi‐do, Korea) on 60‐mm culture dishes. Conditioned MCECs (exposed to glucose conditions with or without rmIGFBP5 or transfection) were seeded into 3‐well blocks at >95% confluence. Five hours later, blocks were removed, and cells were incubated in M199 medium containing 2% FBS and thymidine (2 mM, Sigma‐Aldrich) for 24 h. The images were taken using a phase‐contrast microscope (Olympus), and cell migration was analyzed by determining the percentage of cells that moved into the frame line showed in the figures from four separate block systems in a blinded manner using Image J software (NIH 1.34, http://rsbweb.nih.gov/ij/).

### 2.10 BrdU labeling

BrdU (5′‐bromo‐2′‐deoxyuridine; Sigma‐Aldrich) incorporation assays were performed as described previously.^24^ Conditioned MCECs (exposed to glucose conditions with Sc or siIGFBP5 transfection) were treated with BrdU at a final concentration of 10 μM for 1 h at 37°C, fixed, and subjected to antigen retrieval using 5′‐bromo‐2′‐deoxyuridine antibody (anti‐BrdU, 1:200, Bio‐Rad, Hercules, CA, USA). Images were taken using a confocal fluorescence microscope (K1‐Fluo; Nanoscope Systems, Inc). Numbers of BrdU‐positive endothelial cells were counted at a screen magnification of 200× in four different regions in a blinded manner using Image J software (NIH 1.34, http://rsbweb.nih.gov/ij/).

### 2.11 Aortic ring assay

Aortic ring assays were performed as described previously.^25^, ^26^ Aortas collected from 8‐week‐old C57BL/6 mice were cut into ∼1‐mm thick rings, placed in an 8‐well Nunc Lab‐Tek Chamber Slide System (Sigma‐Aldrich), covered, and fixed with 50 μl Matrigel. Aortic rings were cultured in complement M199 containing normal‐ or HG with or without shCon or shIGFBP5 (5 × 10^4^ IFU/ml culture medium) with medium changes every 2 days for 8 days in a 5% CO_2_ atmosphere at 37°C. Microvessel outgrowths were then assessed using a phase‐contrast microscope (Olympus). Results are expressed as areas of sprouting microvessels (K1‐Fluo; Nanoscope Systems, Inc), which were determined using Image J software (NIH 1.34, http://rsbweb.nih.gov/ij/). Four independent experiments were performed in a blinded manner.

### 2.12 Terminal deoxynucleotidyl transferase‐mediated deoxyuridine triphosphate nick‐end labeling assay

Terminal deoxynucleotidyl transferase‐mediated deoxyuridine triphosphate nick‐end labeling (TUNEL) assays were performed using the ApopTag Fluorescein In Situ Apoptosis Detection Kit (S7160, Chemicon, Temecula, CA, USA). Samples were mounted in a solution (Vector Laboratories Inc., Burlingame, CA, USA) containing DAPI (4,6‐diamidino‐2‐phenylindole, a nuclear stain). Digital images were obtained using a confocal fluorescence microscope (K1‐Fluo; Nanoscope Systems, Inc). Numbers of apoptotic cells were counted at a magnification of 100× using Image J software. Four independent experiments were performed in a blinded manner

### 2.13 Histological examination

For fluorescence examinations, mouse penile tissue was fixed in 4% paraformaldehyde overnight at 4°C, and cell samples were fixed in 4% paraformaldehyde for 15 min at RT. After blocking with 1% BSA (Sigma‐Aldrich) for 1 h at RT, frozen tissue sections (12 μm as thick) or cell samples were incubated with primary antibodies at 4°C overnight. The antibodies used were as follows: mouse monoclonal anti‐IGFBP5 (1:200; Santa Cruz Biotechnology, Inc.), hamster monoclonal anti‐PECAM1 antibody (1:50; Millipore), rabbit polyclonal anti‐NG2 antibody (1:50; Millipore), chicken polyclonal anti‐βIII‐tubulin antibody (1:200; Abcam, Cambridge, MA, USA), rabbit polyclonal anti‐phospho‐eNOS Ser^1177^ antibody (1:100; Invitrogen), rabbit polyclonal NOS1 antibody (nNOS; 1:100; Santa Cruz Biotechnology, Inc.), rabbit polyclonal Occludin antibody (1:100; Invitrogen), mouse monoclonal Claudin5 antibody (1:100; Invitrogen), and rabbit polyclonal LDL‐copper oxidized antibody (1:100; Abcam). After several washes with PBS (Gibco), sections were incubated with donkey anti‐rabbit DyLight 550 (1:200; Abcam), donkey anti‐mouse Alexa Fluor 488 (1:200; Jackson ImmunoResearch Laboratories, West grove, PA, USA), goat anti‐Armenian hamster fluorescein isothiocyanate (1:200; Jackson ImmunoResearch Laboratories), and donkey anti‐chicken rhodamine tetramethylrhodamine secondary antibodies (1:200; Jackson ImmunoResearch Laboratories) for 2 h at RT. Samples were mounted using a solution containing DAPI. Sections or cells were visualized, and images were obtained using a confocal microscope (K1‐Fluo; Nanoscope Systems, Inc.). Quantitative analysis was performed using an image analyzer system (NIH Image J 1.34, http://rsbweb.nih.gov/ij/).

### 2.14 Western blotting

Cells and tissues were lysed in RIPA buffer (Sigma‐Aldrich) supplemented with protease (GenDEPOT, LLC, Katy, TX, USA) and phosphatase (GenDEPOT, LLC) inhibitors. Equal amounts of protein (30 μg per lane) from whole‐cell or tissue lysates were resolved by sodium dodecyl‐sulfate polyacrylamide gel electrophoresis (SDS‐PAGE) on 8% to 12% gels and then transferred to polyvinylidene fluoride membranes. After blocking with 5% nonfat dried milk for 1 h at RT, membranes were incubated at 4°C overnight with the following primary antibodies: anti‐IGFBP5 (1:1000; Santa Cruz Biotechnology, Inc.), anti‐phospho‐eNOS Ser^1177^ (1:500; Cell Signaling), anti‐eNOS (1:1000; Becton Dickinson), anti‐phospho‐AKT (1:1000; Cell Signaling), anti‐AKT (1:1000; Cell Signaling), anti‐phospho‐p38 (1:500; Cell Signaling), anti‐p38 (1:1000; Cell Signaling), anti‐phospho‐ERK (1:1000; Cell Signaling), anti‐ERK (Cat# 9102, Cell Signaling; 1:1000), and anti‐β‐actin (1:4000; Abcam).

IGFBP5 was detected in 10 ml of MCECs cultured conditioned media (culture 80% confluent MCECs in 100 mm dishes for 72 h under NG and HG conditions) by centrifuging at 1500 rpm for 5 min to remove cell debris, then precipitating CM samples with a trichloroacetic acid (TCA) and acetone mixture (10% TCA and 10 mM dithiothreitol [DTT] in acetone, Sigma‐Aldrich) at −20°C overnight.^27^ Precipitated proteins were washed twice with 20 nM DTT in acetone and lysed in RIPA buffer (Sigma‐Aldrich) supplemented with protease inhibitors (GenDEPOT, LLC) and phosphatase inhibitors (GenDEPOT, LLC). Equal amounts of proteins (30 μg per lane) from lysates were resolved by SDS–PAGE and immunoblotted using anti‐IGFBP5 (1:1000; Santa Cruz Biotechnology, Inc.). Membranes were stained using PowerStain Solution (Ponceau S; EPISBIO, Hanam‐si, Gyeonggi‐do, Korea) to confirm equal sample loadings. Densitometric analyses of Western blot bands were performed using an image analyzer system (NIH Image J 1.34, http://rsbweb.nih.gov/ij/).

### 2.15 Statistical analysis

Results are expressed as the means ± SEMs of at least four independent experiments. The unpaired *t* test was used to compare two groups and one‐way ANOVA followed by Tukey's post hoc test for four‐group comparisons. The analysis was conducted using GraphPad Prism version 8 (Graph Pad Software, Inc.), and statistical significance was accepted for *p* values < 0.05.

## 3 RESULTS

### 3.1 Metabolic variables

Body weights of streptozotocin (STZ)‐treated (diabetic) mice were significantly lower than those of age‐matched nondiabetic controls. Fasting and postprandial blood glucose concentrations were significantly higher in diabetic mice than in nondiabetic controls. However, diabetic mice infected with scrambled control shRNA (shCon) lentivirus or IGFBP5 lentivirus had body weights and blood glucose levels similar to those of controls. MSBP were similar in three STZ‐treated groups and the nondiabetic control group (Table S1).

### 3.2 IGFBP5 expression was increased under HG or diabetic conditions

**FIGURE 1:**
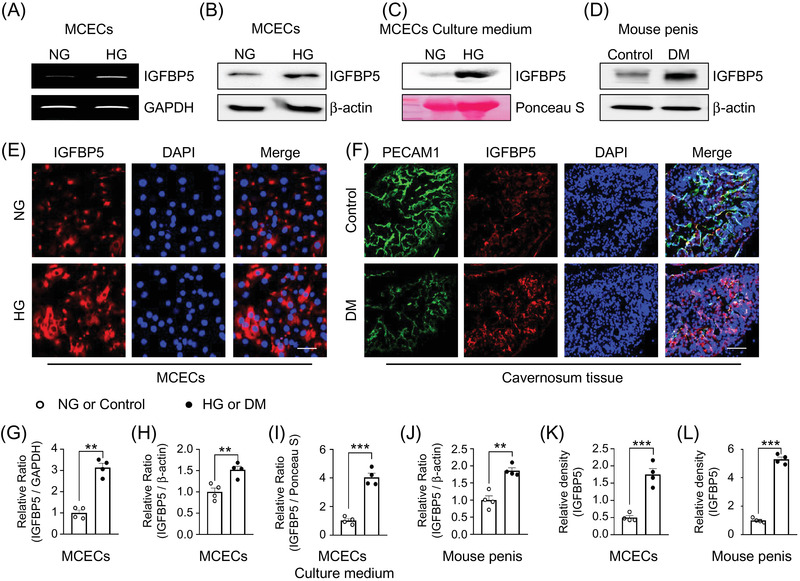
Increased IGFBP5 expression under HG or diabetic conditions. (A) IGFBP5 gene expression was evaluated in MCECs exposed to NG or HG conditions. (B–D) Representative Western blots of IGFBP5 in MCECs and MCEC culture media after exposure to NG or HG conditions in vitro and in age‐matched control and diabetic mouse penile tissue in vivo. (E and F) IGFBP5 (red) or PECAM1 (green, an endothelial cell marker) immunostaining in MCECs and mouse cavernosum tissue. Nuclei were labeled with DAPI. The scale bars indicate 50 μm for MCECs and 100 μm for cavernosum tissue. (G–J) Normalized relative band intensity values as determined using ImageJ (*n* = 4, **p < 0.01, ***p < 0.001). (K) IGFBP5‐immunopositive areas in MCECs under NG or HG condition were quantified using ImageJ (*n* = 4, ***p < 0.001). (L) IGFBP5‐immunopositive areas in PECAM1 expressing cells were quantified using ImageJ (*n* = 4, ***p < 0.001). Data in graphs are presented as means ± SEMs. The value expressed as ratios of the NG or Control group was set to 1. DAPI, 4,6‐diamidino‐2‐phenylindole; DM, STZ‐induced diabetes; HG, high glucose; MCECs, mouse cavernous endothelial cells; NG, normal glucose

IGFBP5 gene expressions were assessed by RT‐PCR in MCECs (Figure S1) exposed to NG or HG conditions. The results obtained showed that IGFBP5 expression was dramatically increased in MCECs exposed to HG (Figures 1A, 1G). In addition, western blot showed IGFBP5 expression was significantly higher in the lysates (Figure 1B,H) and MCEC CM (Figure 1C,I) exposed to HG conditions, and in corpus cavernosum tissues of diabetic mice than in non‐diabetic controls (Figure 1D,J). Also, immunofluorescent staining results confirmed that IGFBP5 expression was significantly higher in MCECs (Figure 1E,K) exposed to HG than NG conditions and higher in the corpus cavernosum tissues of diabetic mice than in nondiabetic controls (Figure 1F,L). These findings provide a rationale for using IGFBP5 blockers to treat diabetic ED.

### 3.3 Knockdown of IGFBP5 induced MCEC angiogenic activity under HG conditions

**FIGURE 2:**
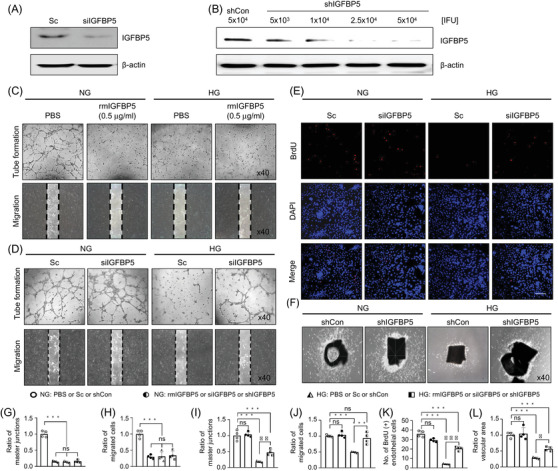
Knockdown of IGFBP5 restored the angiogenic activity of endothelial cells in vitro and ex vivo. (A and B) Representative data for IGFBP5 knockdown by IGFBP5 siRNA transfection or IGFBP5 shRNA lentivirus infection (5 × 10^3^, 1 × 10^4^, 2.5 × 10^4^, or 5 × 10^4^ IFU/ml of culture medium) in mouse cavernous endothelial cells (MCECs). (C and D) Tube formation and migration assays in primary cultured MCECs exposed to NG or HG conditions and treated with rmIGFBP5 protein (0.5 μg/ml), Sc, or siIGFBP5. (E) BrdU incorporation assay of primary cultured MCECs exposed to NG or HG conditions treated with Sc or siIGFBP5. Nuclei were labeled with DAPI (blue). Scale bars indicate 100 μm. (F) Aortic ring assay in mouse aortas exposed to NG or HG conditions and treated with lentiviral particles of shCon or shIGFBP5. (G and I) Numbers of master junctions per field (*n* = 4, **p < 0.01, ***p < 0.001). 40X magnification. (H and J) Migrated cells were quantified using ImageJ (*n* = 4, ***p < 0.001). (K) Numbers of BrdU‐immunopositive endothelial cells were quantified using ImageJ (*n* = 4, ***p < 0.001). (L) Areas of microvessels growing from aortic rings were also quantified using ImageJ (*n* = 4, *p < 0.05, ***p < 0.001). Data in graphs are presented as means ± SEMs. The value expressed as ratios of the NG: PBS, Sc or shCon group was set to 1. BrdU, bromodeoxyuridine; DAPI, 4.6‐diamidino‐2‐phenylindole; IFU, infection units; ns, not significant; Sc, scrambled siRNA; shCon, scrambled shRNA; shRNA, short hairpin RNA; shIGFBP5, shRNA for IGFBP5; siIGFBP5, siRNA for IGFBP5; siRNA, small interfering RNA

To investigate the effects of IGFBP5 expression levels on endothelial cells, we transfected MCECs with siIGFBP5 (Figure 2A) and different doses of shIGFBP5 lentivirus (Figure 2B) to knockdown IGFB5. The angiogenic activities of MCECs were examined after treating cells with recombinant mouse IGFBP5 (rmIGFBP5) protein or IGFB5 knockdown under different glucose conditions. rmIGFBP5 treatment significantly impaired tube formation by MCECs (Figure 2C,G, and Figure S2) and cell migration (Figure 2C,H) under NG conditions, but no significant difference in angiogenic activity was observed between MCECs treated with or without rmIGFBP5 under NG or HG conditions (Figure 2C,G,H). On the other hand, siRNA knockdown of IGFBP5 promoted angiogenic activity under HG conditions but had no significant effect under NG conditions (Figure 2D,I,J). Also, BrdU incorporation assays showed MCEC proliferation was significantly reduced and that the siRNA‐mediated knockdown of IGFBP5 significantly enhanced MCEC proliferation under HG glucose conditions, but that no significant change occurred under NG conditions (Figure 2E,K). In addition, consistent with our in vitro MCEC tube‐formation assay results, microvessel sprouting from aortic rings were severely reduced under HG conditions, and IGFBP5 knockdown with shIGFBP5 lentivirus suppressed this reduction under HG conditions (Figure 2F,L). Overall, these results indicate that the knockdown of IGFBP5 can induce angiogenic activity under HG conditions.

### 3.4 Knockdown of IGFBP5 improved erectile function in diabetic mice

**FIGURE 3:**
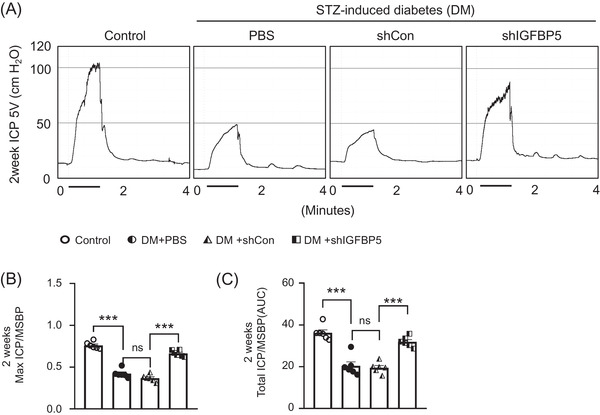
Knockdown of IGFBP5 improved erectile dysfunction in mice with STZ‐induced diabetes (DM). (A) Representative ICP responses for age‐matched nondiabetic controls, diabetic mice stimulated at 2 weeks after an intracavernous PBS, shCon‐, or shIGFBP5‐lentivirus (20 μl for PBS, 5×10^4^ IFU/mouse for shRNA lentiviral particles) injection. The cavernous nerve was stimulated at 5 V, and stimulus time is indicated by a solid bar. (B and C) Ratios of mean maximal ICP and total ICP (area under the curve) versus MSBP were calculated for each group (*n* = 6, ***p < 0.001). Data in graphs are presented as means ± SEMs. DM, STZ‐induced diabetes; ICP, intracavernous pressure; MSBP, mean systolic blood pressure; ns, not significant; shCon, scrambled shRNA; shIGFBP5, shRNA for IGFBP5; shRNA, short hairpin RNA; STZ, streptozotocin;

To investigate whether IGFBP5 knockdown has beneficial effects on erectile function in diabetic mice, we knocked down IGFBP5 with an intracavernous injection of shIGFBP5 lentivirus in diabetic mice and evaluated erectile function 2 weeks later. During electrical stimulation, ratios of maximal and total ICP to MSBP were significantly lower in PBS‐treated and shCon lentivirus infected diabetic mice than in age‐matched nondiabetic controls. Intriguingly, infection of diabetic mice with shIGFBP5 lentivirus significantly improved these erection parameters to almost 90% of control values (Figure 3). However, no detectable differences in MSBP were observed between the three STZ‐induced experimental groups. These results suggest that IGFBP5 knockdown effected diabetes‐induced ED recovery in our mouse model.

### 3.5 Knockdown of IGFBP5 increased endothelial cell, pericyte, and neuronal cell contents in the cavernosum tissues of diabetic mice

**FIGURE 4:**
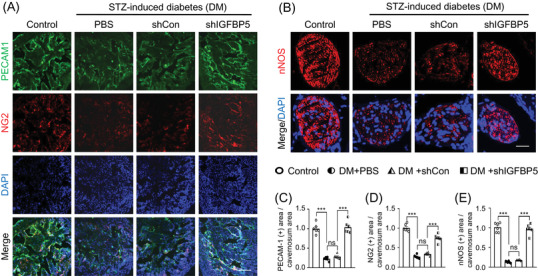
IGFBP5 knockdown increased cavernous endothelial cell, pericyte, and nNOS‐expressing neuronal cell contents in mice with STZ‐induced diabetes (DM). (A) PECAM1 (an endothelial cell marker, green) or NG2 (a pericyte marker, red) immunostaining in cavernous tissues from age‐matched nondiabetic controls and diabetic mice stimulated 2 weeks after intracavernous a PBS, shCon‐, or shIGFBP5‐lentivirus (20 μl for PBS, 5×10^4^ IFU/mice for shRNA lentiviral particles) injection. Scale bar indicates 100 μm. (B) nNOS (a neuronal cell marker, red) immunostaining in the dorsal nerve bundles of an age‐matched nondiabetic control and a diabetic mouse stimulated at 2 weeks after receiving intracavernous PBS, shCon‐, or shIGFBP5‐lentivirus (20 μl for PBS, 5×10^4^ IFU/mice for shRNA lentiviral particles) injections. Nuclei were labeled with DAPI (blue). Scale bar indicates 25 μm. (C–E) Quantifications of cavernous endothelial cells, pericytes, or nNOS‐expressing neuronal cells by ImageJ (*n* = 6, ***p < 0.001). Data in graphs are presented as means ± SEMs. The value expressed as ratios of the Control group was set to 1. DAPI = 4.6‐diamidino‐2‐phenylindole; DM, STZ‐induced diabetes; ns, not significant; shCon, scrambled shRNA; shIGFBP5, shRNA for IGFBP5; shRNA, short hairpin RNA

To determine the effects of IGFBP5 knockdown on neurovascular regeneration in diabetic mice, we performed immunofluorescent staining for cavernous endothelial cells, pericytes, and neuronal cells in cavernosum tissues. We found that the contents of PECAM1‐positive endothelial cells (Figure 4A,C),NG2‐positive pericytes (Figure 4A,D), and nNOS‐positive neuronal cells (Figure 4B,E) were significantly lower in PBS‐treated and shCon lentivirus‐infected diabetic mice than in nondiabetic controls. Interestingly, shIGFBP5 lentivirus infection dramatically promoted these neurovascular contents in diabetic mice (Figure 4). These results indicate that knockdown of IGFBP5 enhances cavernous endothelial cell, pericyte, and neuronal cell contents, which indicates IGFBP5 knockdown might reduce ED in diabetic mice.

### 3.6 Knockdown of IGFBP5 induced eNOS Ser^1177^ phosphorylation and decreased apoptosis of endothelial cells in diabetic mice

**FIGURE 5:**
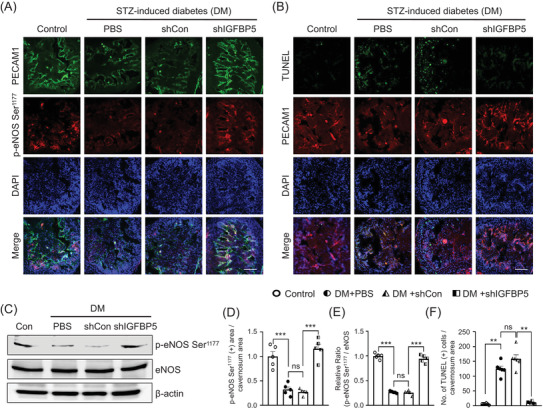
Knockdown of IGFBP5 induced cavernous eNOS Ser^1177^ phosphorylation and reduced cavernous endothelial cell apoptosis in mice with STZ‐induced diabetes (DM). (A) PECAM1 (green) and p‐eNOS Ser^1177^ (red) immunostaining in cavernous tissues from age‐matched nondiabetic control and diabetic mice stimulated at 2 weeks after receiving an intracavernous PBS, shCon‐, or shIGFBP5‐lentivirus (20 μl for PBS, 5 × 10^4^ IFU/mice for shRNA lentiviral particles) injection. The scale bar indicates 100 μm. (B) TUNEL (green) and PECAM1 (red) immunostaining in cavernous tissues from age‐matched nondiabetic controls and diabetic mice stimulated at 2 weeks after receiving an intracavernous PBS, shCon‐, or shIGFBP5‐lentivirus (20 μl for PBS, 5 × 10^4^ IFU/mice for shRNA lentiviral particles) injection. Nuclei were labeled with DAPI (blue). The scale bar indicates 100 μm. (C) Representative Western blots of p‐eNOS Ser^1177^ and eNOS in the control and three experimental groups. (D) Quantitative analysis of p‐eNOS Ser^1177^ immunopositive areas by ImageJ (*n* = 5, ***p < 0.001) (E) Normalized band intensity values for p‐eNOS Ser^1177^ and eNOS were quantified using ImageJ (*n* = 5, ***p < 0.001). The value expressed as ratios of the Control group was set to 1. (F) Number of apoptotic cells were quantified by ImageJ (*n* = 6, ***p < 0.001). Data in graphs are presented as means ± SEMs. DAPI = 4.6‐diamidino‐2‐phenylindole; DM, STZ‐induced diabetes; eNOS, endothelial nitric oxide synthase; NOS, nitric oxide synthase; p‐eNOS, phospho‐endothelial nitric oxide synthase; shCon, scrambled shRNA; shIGFBP5, shRNA for IGFBP5; shRNA, short hairpin RNA; ns, not significant; TUNEL, terminal deoxynucleotidyl transferase‐mediated deoxyuridine triphosphate nick end labeling

Low eNOS phosphorylation levels and endothelial cell apoptosis are the main features of diabetic endothelial dysfunction,^28^ and therefore, we examined the eNOS Ser^1177^ phosphorylation in cavernous tissues by immunofluorescent staining and western blot. eNOS Ser^1177^ phosphorylation was significantly lower in PBS‐treated and shCon lentivirus infected diabetic mice than in age‐matched nondiabetic controls. However, shIGFBP5 lentivirus knockdown of IGFBP5 significantly induced eNOS Ser^1177^ phosphorylation in diabetic mice (Figure 5A,C,D,E). We also found the production of NO was significantly decreased in diabetic conditions compared with control groups in vitro (MCECs cultured medium) and in vivo (penis cavernous tissues); however, siRNA knockdown of IGFBP5 or lentiviral shRNA‐mediated IGFBP5 knockdown significantly promoted the production of NO under diabetic conditions (Figure S3 **)**. Furthermore, TUNEL assays showed that apoptotic endothelial cell numbers were higher in PBS‐treated and shCon lentivirus infected diabetic mice than in age‐matched nondiabetic controls. Moreover, lentiviral shRNA‐mediated IGFBP5 knockdown significantly reduced numbers of apoptotic endothelial cells in the cavernosum of diabetic mice (Figure 5B,F). These results suggest that IGFBP5 knockdown reduces ED by increasing the eNOS Ser^1177^ phosphorylation and decreasing apoptosis in diabetic mice.

### 3.7 Knockdown of IGFBP5 decreased oxidized‐LDL extravasation by restoring endothelial cell‐to‐cell junction protein levels

**FIGURE 6:**
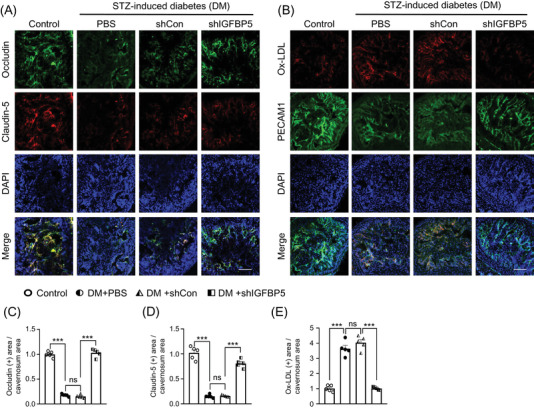
Knockdown of IGFBP5 restored cell‐to‐cell junction protein levels and reduced oxidized LDL extravasation in diabetic mice. (A) Occludin (green) and claudin‐5 (red) immunostaining in cavernous tissues of age‐matched nondiabetic controls and diabetic mice stimulated at 2 weeks after receiving an intracavernous PBS, shCon‐, or shIGFBP5‐lentivirus (20 μl for PBS, 5 × 10^4^ IFU/mice for shRNA lentiviral particles) injection. The scale bar indicates 100 μm. (B) Ox‐LDL (red) and PECAM1 (green) immunostaining in cavernous tissues from age‐matched non‐diabetic controls and diabetic mice stimulated 2 weeks after receiving an intracavernous PBS, shCon‐, or shIGFBP5‐lentivirus (20 μl for PBS, 5 × 10^4^ IFU/mice for shRNA lentiviral particles) injection. The scale bar indicates 100 μm. Nuclei were labeled with DAPI (blue). (C and D) Quantitative analysis of occludin and claudin‐5 immunopositive areas as determined by ImageJ (*n* = 5, ***p < 0.001). (E) Quantitative analysis of Ox‐LDL immunopositive areas by ImageJ (*n* = 5, ***p < 0.001). Ratios are expressed versus non‐diabetic controls. Data in graphs are presented as means ± SEMs. DAPI = 4.6‐diamidino‐2‐phenylindole; DM, STZ‐induced diabetes; ns, not significant; Ox‐LDL, oxidized low‐density lipoprotein; shCon, scrambled shRNA; shIGFBP5, shRNA for IGFBP5; shRNA, short hairpin RNA

To evaluate the expressions of endothelial cell‐to‐cell junction proteins in cavernosum tissues, we performed immunofluorescent staining for occludin and claudin‐5 in diabetic mice. The expressions of occludin and claudin‐5 were lower in PBS‐treated and shCon lentivirus‐infected diabetic mice than in nondiabetic controls, but lentiviral shRNA‐mediated IGFBP5 knockdown significantly restored the levels of these endothelial cell‐to‐cell junction proteins in the cavernosum tissues of diabetic mice (Figure 6A,C,D). In addition, double staining of cavernosum tissues with PECAM1 and oxidized LDL antibodies showed that PBS‐treated and shCon lentivirus‐infected diabetic mice had significantly higher extravasation levels of oxidized LDL than age‐matched nondiabetic controls. However, lentiviral shRNA‐mediated IGFBP5 knockdown significantly decreased this leakage of oxidized LDL in diabetic mice (Figure 6B,E). These results suggest IGFBP5 knockdown reduces the permeability of cavernosum vessels in diabetic mice.

### 3.8 Knockdown of IGFBP5 enhanced activations of the AKT and ERK pathways and inhibited the p38 pathway in diabetic mice

**FIGURE 7:**
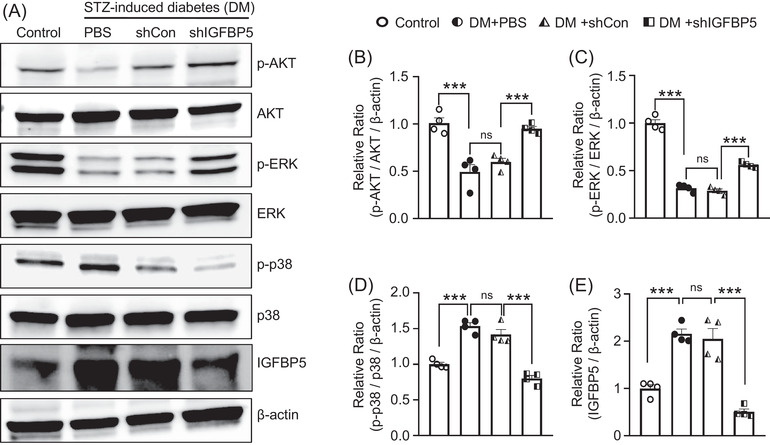
Knockdown of IGFBP5 enhanced AKT and ERK signaling and inhibited p38 signaling in diabetic mice. (A) Representative western blots for phospho‐AKT/AKT, phospho‐ERK/ERK, phospho‐p38/p38, and IGFBP5 in cavernous tissues from nondiabetic controls and diabetic mice stimulated 2 weeks after receiving an intracavernous PBS, shCon‐, or shIGFBP5‐lentivirus (20 μl for PBS, 5 × 10^4^ IFU/mice for shRNA lentiviral particles) injection. (B and E) Data are presented as relative intensities of p‐AKT, p‐ERK, p‐p38, and IGFBP5 proteins versus AKT, ERK, p38, and β‐actin, respectively, as determined by ImageJ (*n* = 4, ***p < 0.001). The value expressed as ratios of the Control group was set to 1. Data in graphs are presented as means ± SEMs. DM, STZ‐induced diabetes; ns, not significant; shCon, scrambled shRNA; shIGFBP5, shRNA for IGFBP5; shRNA, short hairpin RNA

To determine the mechanism responsible for inhibition of the angiogenesis signaling pathway by IGFBP5, we examined cell survival‐related signaling (AKT and ERK) and apoptosis‐related signaling (p38) after IGFBP5 knockdown in diabetic mice. Interestingly, the phosphorylations of AKT and ERK were lower in PBS‐treated and shCon lentivirus infected diabetic mice than in nondiabetic controls. Lentiviral shRNA‐mediated knockdown of IGFBP5 significantly enhanced the phosphorylations of AKT and ERK (Figure 7A,B,C) but reduced diabetes‐induced increases in p38 phosphorylation and IGFBP5 expression (Figure 7A,D,E). These results indicate that mediations of the AKT, ERK, and p38 signaling pathways by IGFBP5 importantly influence the pathogenesis of ED in diabetic mice.

## 4 DISCUSSION

Recently, various angiogenic and neurotrophic factors, such as VEGF, COMP‐Ang1, dickkopf2, and brain‐derived neurotrophic factor, have been subjected to clinical trials for ED but with limited success. Therefore, more candidate molecules are needed to promote angiogenesis and nerve regeneration in diabetic‐induced ED. The current study shows IGFBP5 is a good target for diabetic ED research because its expression in mouse cavernous endothelial cells (MCECs) is significantly increased under HG conditions. IGFBP5 is a 28.5 kDa protein with 252 residues, is expressed and secreted by a variety of tissues and cell types, and also has nuclear activity. The present study shows IGFBP5 is expressed in MCECs, released to culture media, and expressed in mouse penile tissues, and that its protein expression is significantly increased under HG or diabetic conditions. It has been reported that IGFBP5 by acting as an anti‐angiogenic factor, prevents tumor growth and inhibits tumor vascularity in human ovarian cancer.^29^ Therefore, we hypothesized that elevated IGFBP5 expression may be related to the angiopathy observed in diabetic ED, and that better knowledge of the activities of IGFBP5 might provide important clues for the treatment of diabetic ED.

When we exogenously applied mouse IGFBP5 recombinant protein to MCECs, it significantly reduced capillary‐like structures and MCEC migration under NG and HG conditions, whereas siRNA knockdown IGFBP5 significantly promoted angiogenesis under HG conditions. In addition, our experimental results showed that IGFBP5 knockdown significantly induced MCECs proliferation and aortic ring microvessel sprouting under HG conditions. These findings indicate that IGFBP5 suppresses MCEC angiogenesis. Nonetheless, it is not known whether IGFBP5 is released by other cell types under other pathological conditions, and the detailed mechanism responsible for the effects of IGFBP5 on angiogenesis remain to be elucidated.

Members of the IGFBP family regulate cell survival, migration, senescence, angiogenesis, and apoptosis, and thus, they participate in many physiological and pathological processes, including immune regulation, cancer, and neurological diseases.^30^ Firth et al. showed IGFBP‐1, ‐2, ‐3, and ‐5 may also enhance IGF activity.^31^ Recently, many studies have reported that shRNA targeting IGFBP3 improves erectile function by restoring IGF‐1 bioavailability or increasing the concentration of cavernous cGMP in diabetic or age‐related ED rat models.^32^, ^33^, ^34^ In addition, Pu et al. showed that increasing the expression of IGF‐1 can restore erectile function in diabetic or aged rats.^35^, ^36^ However, little research has been performed on the role of IGFBP5 in diabetic ED. We hypothesized that reduced IGFBP5‐mediated signaling might have a beneficial effect on diabetic ED. Our results that knockdown of IGFBP5 in diabetic mice using shIGFBP5 lentiviral particles enhances cavernous endothelial cell, pericyte, and neuronal cell numbers, reduces apoptosis and permeability, and restores erectile function. These results supports our hypothesis. Furthermore, it has been reported that silencing IGFBP5 with small interfering RNA can promote proliferation via AKT and ERK^37^ and reduce apoptosis via p38 in different cell types.^38^ Similarly, we observed IGFBP5 knockdown enhanced these signals to normal levels in diabetic mice. Taken together, these findings indicate that diabetic conditions increase IGFBP5 expression, and that this increase is positively related to diabetes mediated ED. However, the current study does not prove that this effect of IGFBP5 is IGF‐dependent. Nonetheless, we hope this study provides a basis for further study of the therapeutic value of inhibiting IGFBP5 in diabetic ED.

In summary, our findings show that knockdown of IGFBP5 expression in erectile tissues restored damaged vascular by promoting endothelial cell proliferation and reducing apoptosis. To the best of our knowledge, this study is the first to report that IGFBP5 knockdown can improve neurovascular regeneration in diabetic ED. Nevertheless, it has several limitations. First, we did not use L‐glucose as an osmolality control. Second, although we demonstrated that IGFBP5 expression is increased in MCECs under HG conditions, we did not evaluate whether IGFBP5 expression is also increased in other cell types in diabetic ED cavernosum tissues. Third, we only evaluated a limited number of known IGFBP5 signaling pathways in diabetic ED mice. Fourth, the measurement of sprouting microvessels area in the aortic ring assay has certain limitations. Further research is needed to evaluate the detailed mechanisms and functions of IGFBP5 in other vascular or neurogenic ED.

## AUTHOR CONTRIBUTIONS

OJ, YGN, SJK, and RJK designed the study. YGN prepared the STZ‐induced diabetic model, lentivirus infections, and in vitro treatment. OJ and YGN performed all other studies. OJ performed statistical analysis. OJ and YGN wrote the manuscript. YGN, HSS, KJH, and RJK received grants. SJK and RJK supervised the whole study. All authors reviewed the manuscript and approved the final version.

## CONFLICT OF INTEREST

The authors declare no conflict of interest.

## Supporting information

Supporting Information

## Data Availability

The data that support the findings of this study are available from the corresponding author upon reasonable request.
