# Supplementary material for: IGFBP5 antisense and short hairpin RNA (shRNA) constructs improve erectile function by inducing cavernosum angiogenesis in diabetic mice
Source: Andrology. 2022 Aug 7;11(2):358–71. doi: 10.1111/andr.13234 (PMC10087557; doi:10.1111/andr.13234)
Supplement: Supplementary file 1 — Supporting Information [file ANDR-11-358-s001.docx]

**Supplementary informations**

**IGFBP5 antisense and short hairpin RNA (shRNA) constructs improve erectile function by inducing cavernosum angiogenesis in diabetic mice**

Jiyeon Ock ^1^, Jun-Kyu Suh^1^, Soon-Sun Hong^2^, Ju-Hee Kang^3^, Guo Nan Yin^1^, Ji-Kan Ryu^1^

^
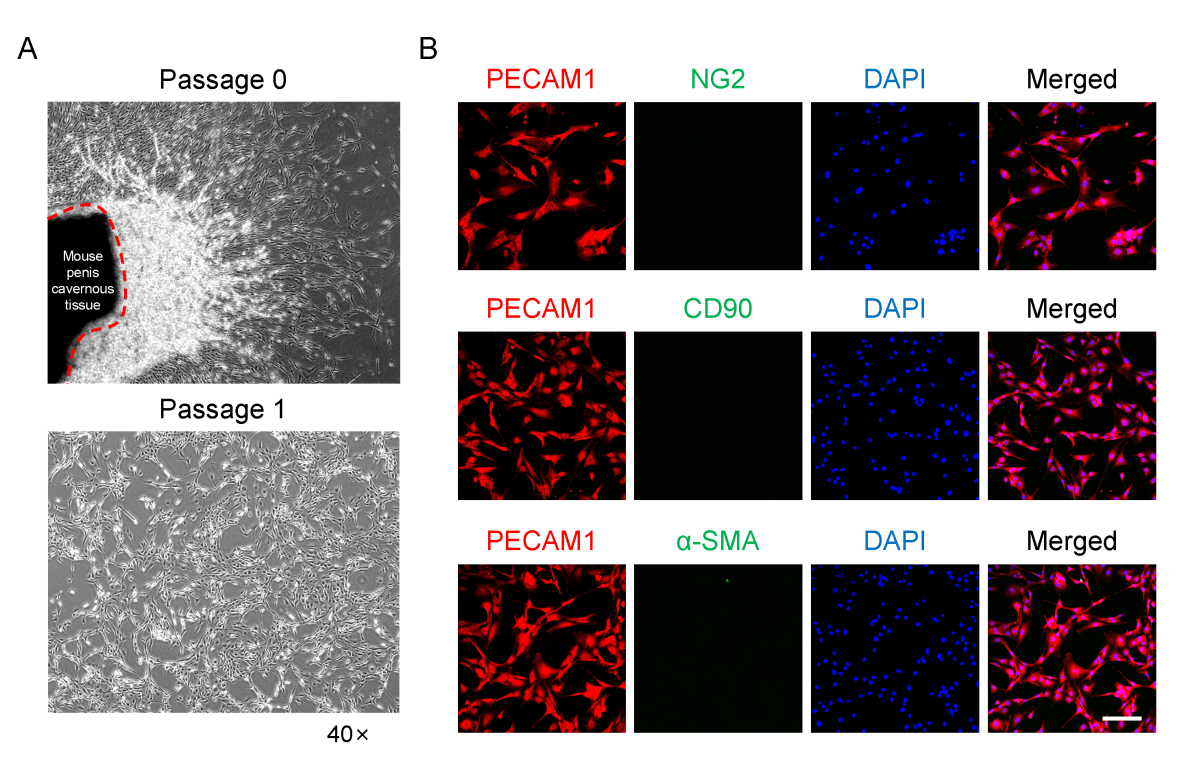
^

**Supplementary Figure S1**. Isolation and characterization of primary cultured mouse cavernous endothelial cells (MCECs). (A) Representative phase-contrast image (screen magnification, 40×) of sprouted cells from mouse penis cavernous tissues at passage 0 and sub-cultured passage 1. (B) Immunofluorescent staining of sprouted cells with antibodies against PECAM1 (an endothelial cell positive marker), NG2 (a pericytes marker), CD90 (a fibroblast marker), and α-SMA (a smooth muscle cell marker). Nuclei were labeled with the DNA dye DAPI. Scale bar indicates 100 μm. DAPI = 4,6-diamidino-2-phenylindole.

^
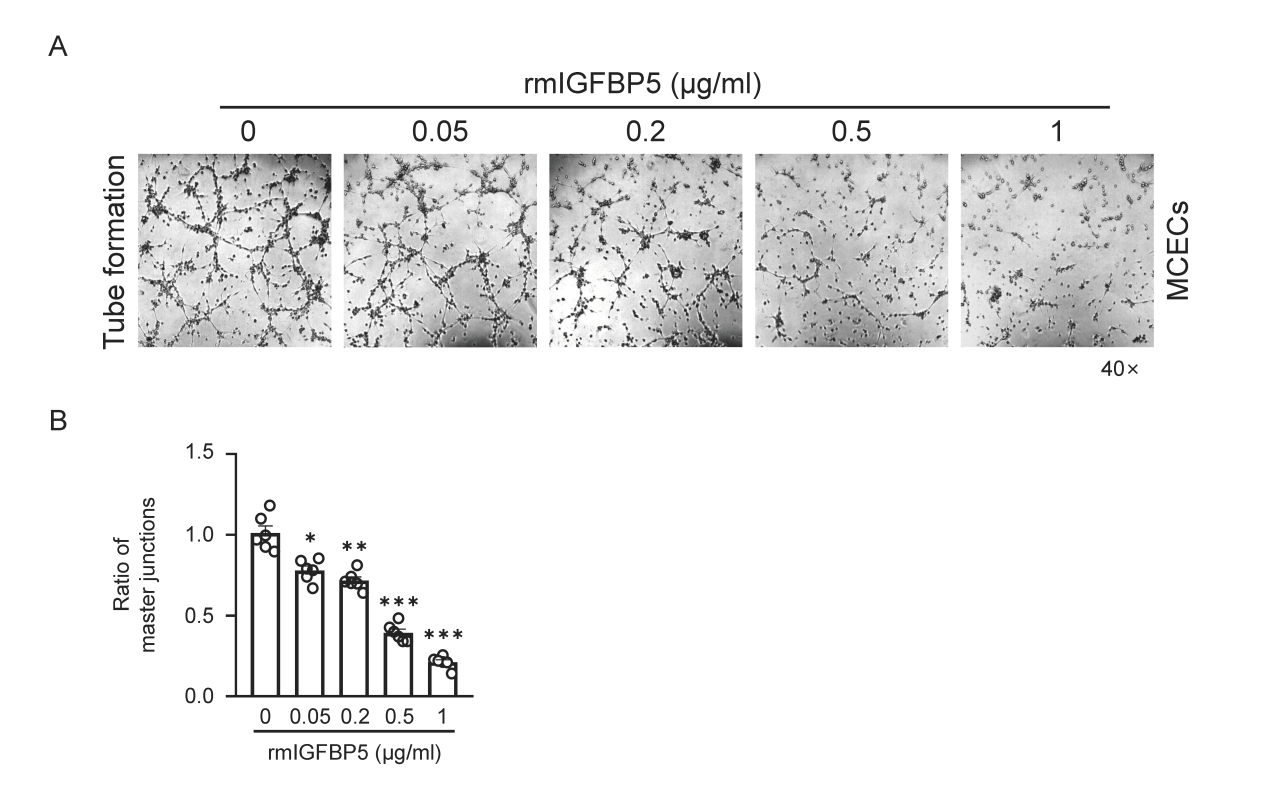
^

**Supplementary Figure S2**. IGFBP5 decreased mouse cavernous endothelial cells (MCECs) tube formation in a dependent manner. (A) Tube formation assay by MCECs treated with recombinant mouse IGFBP5 protein (rmIGFBP5, 0, 0.05, 0.2, 0.5, 1 µg/ml) for 24 hours. Phase-contrast images were acquired 16 hours after plating (screen magnification, 40×). (A) The number of master junctions was quantified using Image J, and the results are presented as means ± SEMs (n = 6; **P* < 0.05, ***P* < 0.01; ****P* < 0.001). The relative ratio in the 0 µg/ml group was set to 1.

^
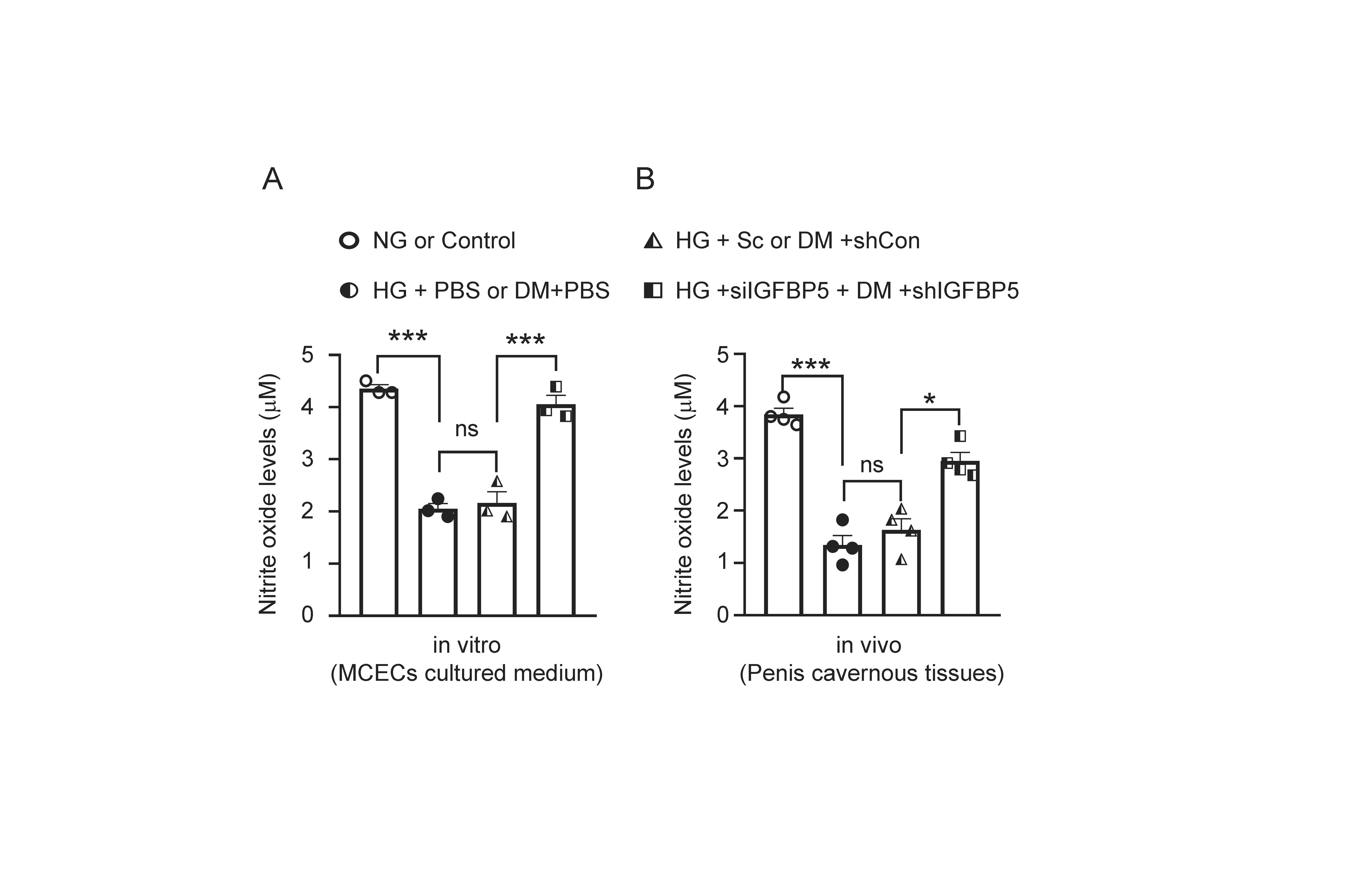
^

**Supplementary Figure S3.** Knockdown of IGFBP5 induced nitrite oxide production in diabetic conditions *in vitro* (MCECs cultured medium) and *in vivo* (penis cavernous tissues). (A) Nitrite oxide levels in cultured medium of MCECs, which exposed to NG or HG conditions with PBS, scrambled control (Sc)-, or siIGFBP5-transfection for 72 hours. Data in graphs are presented as means±SEMs (n =3, ****P*< 0.001). (B) Nitrite oxide levels in penis cavernous tissues from age-matched non-diabetic control and diabetic mice receiving an intracavernous PBS, shCon-, or shIGFBP5-lentivirus (20 µL for PBS, 5 x10^4^ IFU/mice for shRNA lentiviral particles) injection for 2 weeks. Data in graphs are presented as means±SEMs (n = 4, **P*< 0.05, ****P*< 0.001). NG, normal glucose; HG, high glucose; DM, STZ-induced diabetes; siIGFBP5, small interfering RNA for IGFBP5; shCon, scrambled short hairpin RNA; shIGFBP5, short hairpin RNA for IGFBP5; ns, not significant.

| **Supplementary Table S1.** Physiologic and metabolic parameters: 2 weeks after treatment with PBS, shCon, shIGFBP5. | | | | |
| --- | --- | --- | --- | --- |
|  | | STZ-induced diabetic mice | | |
|  | Control | PBS | shCon | shIGFBP5 |
| Body weight (g) | 26.7±0.9 | 20.4±1.2* | 20.7±0.9* | 20.3±1.1* |
| Fasting glucose (mg/dl) | 102.9±4.4 | 520.4±57.5* | 494.6±69.2* | 493.6±62.8* |
| Postprandial glucose (mg/dl) | 156.5±17.4 | 594.1±15.3* | 575.0±53.8* | 570.4±40.2* |
| MSBP (mm Hg) | 96.7±6.0 | 100.9±9.9 | 97.6±6.4 | 98.8±7.0 |
| Values are the mean±SEM for n=20 animals for control group, n=18 animals for DM+PBS group, n=16 animals for DM+shCon group, n=16 animals for DM+shIGFBP5 group. STZ, streptozotocin; MSBP,Mean systolic blood pressure; shCon, scrambled shRNA; shIGFBP5, shRNA for IGFBP5; shRNA, short hairpin RNA **P* < 0.05 vs. Control group | | | | |
